# Computational Fluid Dynamics Study of Erosion on 900 MW Steam Turbine ND-45 Blades Using 3D Scanning

**DOI:** 10.3390/ma17194884

**Published:** 2024-10-04

**Authors:** Grzegorz Bzymek, Mateusz Bryk, Sylwia Kruk-Gotzman, Piotr Józef Ziółkowski

**Affiliations:** 1 Institute of Fluid-Flow Machinery, Polish Academy of Sciences, 80-231 Gdańsk, Poland; grzegorz.bzymek@gkpge.pl (G.B.);; 2 PGE GiEK S.A. Branch Office Power Plant Opole, 45-920 Opole, Poland

**Keywords:** 3D scanning, CFD calculations, erosion, steam turbine, ND-45 blade, X4CrNiCuMo14-5

## Abstract

This paper presents a comprehensive study on the impact of erosion on the flow characteristics through the blade of the last stage of a 900 MW steam turbine. The primary objective is to understand how surface erosion, caused by prolonged steam exposure, affects flow behavior and the overall efficiency of a 900 MW class turbine. The research process began with a 3D scan of the turbine blade, using advanced laser scanning technology to create a detailed geometric model. As one of the longest blades used in steam turbines, it posed both a technical challenge and was an innovative aspect of this study. The resulting 3D model served as the basis for numerical simulations using Computational Fluid Dynamic (CFD) methods, which allowed for the analysis of steam flow over the eroded blade surface. Key flow parameters, including velocity, pressure, and turbulence, were assessed to determine the impact of erosion. The study revealed significant changes in flow characteristics depending on the degree of erosion, providing valuable insights for turbine optimization and maintenance. The novelty of this research lies not only in the use of advanced scanning technologies but also in analyzing one of the longest blades in industrial practice, with findings that could enhance turbine efficiency and inform new erosion risk management strategies.

## 1. Introduction

The erosion of steam turbine blades is a critical issue that impacts the performance, longevity, and reliability of power generation systems. Even with the continuous advancement of materials science and surface protection technologies, erosion remains a persistent challenge, especially in high-pressure and high-temperature environments where turbines operate. In particular, turbine blades are subjected to various erosive agents, including steam, solid particles, and water droplets. These agents contribute to material degradation over time, causing efficiency losses, increased fuel consumption, and maintenance costs [1]. While the application of protective coatings has shown some success in mitigating erosion, research indicates that the protective effect is strongly dependent on parameters such as coating thickness, test temperature, and coating hardness [2]. The selection and optimization of these parameters are essential for enhancing the erosion resistance of turbine blades.

The formation of erosion on turbine blades is predominantly observed on the leading edge, which is the first point of impact for particles and water droplets. Studies have shown that erosion tends to occur more severely in specific zones, such as behind secondary droplet generation areas [3,4]. These droplets form due to vapor flow detachment in regions of high turbulence and secondary flow, further accelerating erosion in downstream stages [5]. The material loss resulting from this process alters the blade geometry and surface roughness, leading to significant changes in the aerodynamic performance of the turbine. Surface roughness caused by erosion increases boundary layer thickness, decreases the efficiency of steam flow, and ultimately leads to higher energy losses within the turbine [6,7]. Thus, surface roughness is a key factor in determining the overall impact of erosion on turbine performance.

In response to this, several protective measures have been proposed to reduce the effects of erosion, particularly at the leading edge of turbine blades. One such approach is laser hardening, which aims to increase the durability of the blade’s leading edge by creating a hardened surface layer. However, errors in the hardening process, such as insufficient hardened bandwidth, can lead to early-stage failures [8]. Moreover, studies have highlighted the importance of optimizing the blade’s leading-edge geometry, which plays a critical role in determining the blade’s resistance to erosive forces [9]. Projecting blades, for example, tend to experience more severe erosion than their retracted counterparts, which has prompted further investigations into the aerodynamic effects of blade position and profile in relation to erosion [10].

The indirect effects of erosion are equally concerning. Beyond material degradation, erosion can lead to changes in the blade’s mechanical properties, such as kinetostatic stress fields, dynamic characteristics, and fatigue life [11,12,13]. These factors significantly affect the overall performance and durability of the turbine, emphasizing the need for comprehensive monitoring and diagnostic strategies. The development of advanced diagnostic tools, such as laser stripe inspection and 3D scanning, has opened up new possibilities for assessing erosion damage with unprecedented precision [6]. These tools not only enable the more accurate measurement of surface degradation but also provide valuable insights into the underlying causes of erosion and its progression over time.

Recent research has also focused on understanding the mechanisms of erosion caused by different types of particles, such as water droplets and solid contaminants. Water droplet erosion, in particular, has been identified as a major concern in low-pressure stages of steam turbines, where condensation occurs. Numerical models have been developed to predict the impact of water droplet erosion on turbine blades, providing a framework for designing blades that are more resistant to such damage [14,15]. Solid particle erosion, on the other hand, tends to be more prevalent in high-pressure stages, where particles suspended in steam impact the blade’s surface at high velocities. Predictive equations and experimental studies have been used to estimate the damage caused by these particles, leading to the development of more robust erosion-resistant coatings and materials [2,16].

The effects of surface roughness on the aerodynamic performance of turbine blades have been widely studied. Roughness not only increases energy losses but also influences the behavior of the boundary layer, which directly impacts the turbine’s overall efficiency. Measurements on low-pressure turbine blades have shown that even small changes in surface roughness can lead to significant variations in aerodynamic loading and boundary layer development [7,17]. This is particularly important for the optimization of blade design, as even minor improvements in surface smoothness can lead to noticeable gains in performance.

This paper presents an in-depth analysis of erosion in the final-stage blades of a 900 MW steam turbine (model ND-45), constructed from X4CrNiCuMo14-5 steel. This study leverages advanced 3D scanning technology to model the geometry of one of the longest blades used in steam turbines, enabling the precise evaluation of the effects of erosion on steam flow dynamics. This research is notable for its integration of modern diagnostic techniques, which provide a new perspective on turbine blade erosion. Additionally, this study investigates the broader implications of erosion on turbine performance, exploring how changes in blade geometry, surface roughness, and material properties affect the aerodynamic behavior of steam flow through the turbine. The findings of this study have important implications for improving the efficiency, reliability, and longevity of steam turbines in modern power systems.

One of the key innovations in this research is the use of advanced 3D scanning technology to create highly accurate geometric models of turbine blades. As mentioned in the introduction, the use of the Revopoint POP 2 scanner with a precision of 0.05 mm represents a substantial improvement in its ability to capture minute details of blade erosion, including surface roughness and geometric changes over time. Previous studies, such as those by Jiang et al. (2020), explored the application of laser stripe extraction for turbine blade inspection [6]. However, this research goes further by applying 3D scanning to model one of the longest blades used in steam turbines, which is a significant technical challenge. By incorporating this advanced scanning method, the study allows for more accurate representation and quantification of erosion, setting it apart from earlier methods.

While previous studies, such as those by Zhou et al. (2008) [15], provided numerical frameworks for analyzing water droplet erosion on turbine blades, this research advances the field by integrating Computational Fluid Dynamic (CFD) simulations with real-world, high-precision erosion data obtained from 3D scans. This coupling of experimental 3D data with numerical models offers a more detailed and realistic assessment of the impact of erosion on steam flow characteristics. The novelty lies in how these simulations reveal the turbulence, flow separation, and aerodynamic losses caused by the altered blade geometry due to erosion. This combination of techniques is fully explored in earlier research, which often relied on theoretical models or less accurate scanning techniques.

The research emphasizes the critical role of leading-edge geometry and surface roughness in determining the extent of performance loss due to erosion. Studies like those by Bai et al. (2014) [9] and Montis et al. (2010, 2011) [7,17] explored the aerodynamic effects of surface roughness on turbine blades, showing how roughness can increase drag and degrade turbine performance. However, this study adds to existing knowledge by specifically analyzing the leading edge of one of the largest turbine blades in operation, offering new insights into how localized erosion affects steam flow dynamics. By showing how surface irregularities in these regions exacerbate flow disturbances, the research provides a more detailed picture of erosion’s impact beyond what was previously covered in more generalized aerodynamic studies.

Unlike many previous studies that are either theoretical or conducted under controlled laboratory conditions, this research is grounded in practical applications. The turbine blades studied are from a 900 MW industrial steam turbine (ND-45), which is representative of real-world power plant operations. This practical focus enhances the study’s relevance to engineers and operators who deal with erosion management in power plants. By offering concrete findings on how erosion impacts steam flow and overall turbine performance, this research contributes directly to the development of more effective maintenance strategies and the optimization of turbine operations.

The detailed analysis of flow parameters such as velocity, temperature, and turbulent kinetic energy across multiple planes of the turbine blade is another innovative aspect of this study. Previous research, including the work of Oka et al. (2005) [14], provided predictive equations for estimating erosion damage from particle impacts. However, this research builds on such foundational work by linking erosion-induced changes in blade geometry to specific changes in fluid dynamics within the turbine stage. This provides a more holistic understanding of how erosion affects turbine efficiency, particularly in terms of energy losses and flow disturbances.

**Figure 1:**
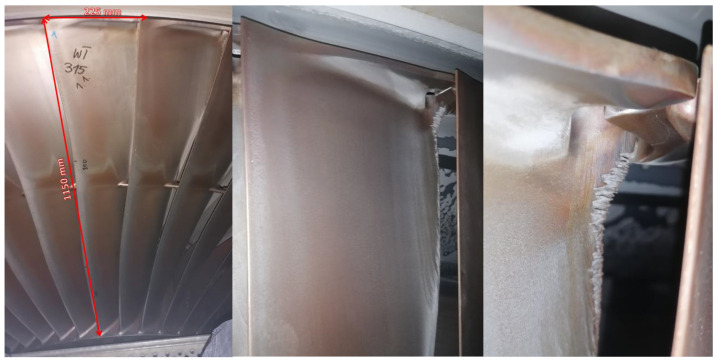
View of the analyzed profile with close-up on the erosion of the trailing edge.

The effects of erosion on the last stage of the analyzed turbine are shown in Figure 1.

The erosion on the run-off surface of last-stage rotor blades does not require immediate intervention, but its impact on the flow should be noticeable, so we decided to focus on this aspect.

## 2. Materials and Methods

### 2.1. X4CrNiCuMo14-5 Steel

X4CrNiCuMo14-5 is a martensitic stainless steel alloy, also known as 1.4542 or 15-5 PH (Precipitation Hardening) stainless steel. It is widely used in high-stress applications, such as turbine blades, due to its excellent combination of mechanical properties, corrosion resistance, and heat treatability. Table 1 contains the chemical composition of the analyzed steel.

Below is a detailed characterization of X4CrNiCuMo14-5 steel as a material for the last-stage turbine blade.

X4CrNiCuMo14-5 steel is an excellent material for last-stage turbine blades, where high mechanical strength, good fatigue resistance, and reliable performance at elevated temperatures are critical. Its corrosion resistance also makes it suitable for the harsh, high-moisture environment typical of steam turbines. These properties make it a preferred choice for high-performance turbine applications where material reliability and longevity are paramount.

### 2.2. Three-dimensional Scanning Method

The Revopoint Pop 2 scanner was used for 3D scanning and can operate as a handheld scanner or function on a tripod with the included rotating platform. The Revopoint POP 2 is equipped with a projector and IR cameras with an improved calibration method to ensure scanning with a precision of 0.05 mm. The point distance is 0.15 mm. The POP 2’s scanning speed is 10 fps. The device is equipped with a 6DoF gyroscope and RGB exposure sensors, which significantly improves the quality of scanned models and also allows the scanning of colored objects. The scanning accuracy with the POP 2 is as high as 0.05 mm [19].

Due to the nature of the object to be scanned (metal parts) as well as the scanning conditions (inside the diffuser), the only acceptable form of scanning was with markers. Markers are circular markers that are stuck onto the scanned object so that the 3D scanner can find reference points on its surface.

**Figure 2:**
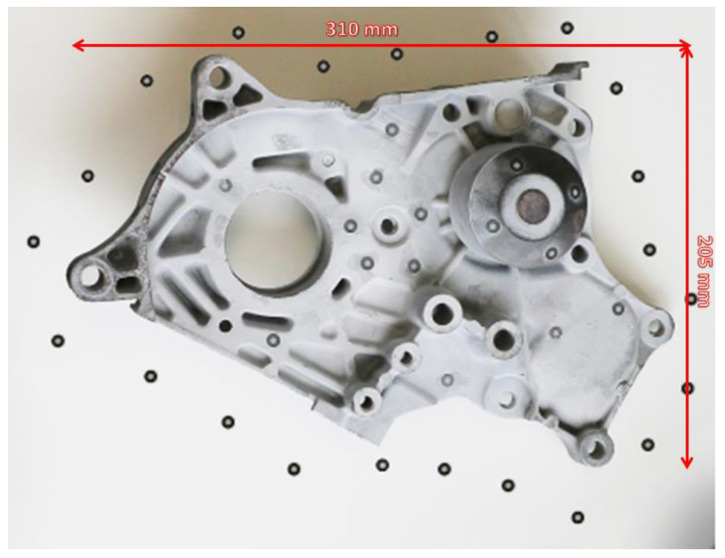
Example of geometry marking for 3D scanning [20].

Markers had to be placed around the scanned object and on irregular parts of the object to maximize the accuracy of the scan. To this end, 3D markers were applied to the rotor blade and inner fuselage. An example of an object with stick-on markers is shown in Figure 2.

### 2.3. CFD Model

In a conservative formulation of the principle of conservation of a given quantity *ϕ*, we consider an arbitrary control volume Ω, located stationary in space, bounded by a surface *∂*Ω. The conservation principle considers that the total change in a given quantity *ϕ*, inside the control volume, is equal to the quantity of the given quantity penetrating the boundary **F**(*ϕ*), called flux, and the production/destruction *S* of the given quantity, which is given by Equation (1) [21].

(1) $$\frac{\partial }{\partial t}\int_{\Omega }\phi d\Omega =-\oint_{\partial \Omega }F\cdot \vec{n}dS+\int_{\Omega }Sd\Omega$$

where

- *dS* is a surface element with normal vector $\vec{n}$;
- *ϕ* is an arbitrary quantity, i.e., a scalar or a vector, then flux;
- **F**—flux—is a vector or tensor, respectively.

The **F**-stream can be divided into the following [22]:

- A convective part $F^{C}\left(\phi \right)=\phi \vec{U}$—related to the transport of a given quantity through the macroscopic movement of a fluid at a speed of $\vec{U}$;
- The diffusion part $F^{D}\left(\phi \right)$—which represents the macroscopic effect of the transport of a given quantity by the chaotic movement of particles;
- The elastic part $F^{V}(\phi )$—related to the deformation of the elastic structure under the influence of fluid forces.

#### 2.3.1. Conservation of Mass Equation

For single-phase fluid, the equation of conservation of mass in Eulerian form takes the notation (2) [23]:

(2) $$\frac{\partial }{\partial t}\int_{\Omega }\phi d\Omega =-\oint_{S}\left(\rho \vec{U}\right)\cdot \vec{n}dS=0$$

I represents the change in mass over time inside the control volume under consideration, which must be equal to the mass over the edge of the control volume in question.

#### 2.3.2. Conservation of Momentum Equation

The change in momentum of fluid volume is caused by a pulse of forces acting on it. Thus, for stationary control volume in space, the total change in momentum, which can be written as the sum of the change in momentum inside the controlled volume and the momentum crossing its boundary, is equal to the forces acting on the considered control volume of the fluid: surface and mass (external) forces. This can be written as follows (3) [21,24]:

(3) $$\frac{\partial }{\partial t}\int_{\Omega }\rho \vec{U}d\Omega +\oint_{d\Omega }\left[\left(\rho \vec{U}\right)⊕\vec{U}\right]\cdot \vec{n}dS=\oint_{d\Omega }\overset{̿}{f_{s}}\cdot \vec{n}dS+\int_{\Omega }\rho \vec{f_{e}}d\Omega$$

where

- $\vec{f_{e}}$—Mass forces, which include gravitational force, Coriolis force, centrifugal force, etc.;
- $\overset{̿}{f_{s}}$—the surface forces acting directly on the edge of the control volume originate from the pressure prevailing around the fluid element under consideration and from the normal and tangential stresses and tangential stresses due to viscous forces, as given by Equation (4):
  (4) $$\overset{̿}{f_{s}}=-p\overset{̿}{I}+\overset{̿}{\tau }$$
  where $\overset{̿}{I}$ is a unit tensor of second order, while $\overset{̿}{\tau }$ is referred to as the viscous stress tensor.

#### 2.3.3. Energy Conservation Equation

The total change in internal energy of a fluid flowing through a non-mobile control volume is understood to be the change in energy within the control volume and the change in energy through transport with the fluid through its edge. This should be taken into account, additionally considering the representation of the heat exchanged by the fluid element as follows (5) [25,26]:

(5) $$\frac{\partial }{\partial t}\int_{\Omega }\rho Ed\Omega \;+\oint_{\partial \Omega }\left(\rho E\vec{U}\right)\cdot \vec{n}dS=\;=\oint_{\partial \Omega }\left(k\nabla T\right)\cdot \vec{n}dS+\int_{\Omega }\rho q_{h}d\Omega -\oint_{\partial \Omega }\left(\rho \vec{U}\right)\cdot \vec{n}dS+\;+\oint_{\partial \Omega }\left(\overset{̿}{\tau }\cdot \vec{U}\right)\cdot \vec{n}dS+\oint_{\partial \Omega }\rho \vec{f_{e}}\cdot \vec{U}d\Omega$$

where

- $\vec{q_{D}}=-\overset{̿}{k}\cdot \nabla T$—diffusive heat flux in which $\overset{̿}{k}$ is the tensor of thermal conductivity;
- $q_{h}$—heat sources representing radiative heat transfer and heat sources from chemical reactions.

#### 2.3.4. Turbulence Model

In this paper, the *k*-ω SST (Shear-Stress Transport) turbulence model is used because it combines the advantages of the *k*-ϵ and *k*-ω models and introduces an additional limiting term for the overproduction of kinetic energy and turbulence in areas of strong positive pressure gradients.

The *k*-ω SST turbulence model is a popular model used to simulate turbulent flow. Here, the general form of the equations for this model is given in Eulerian notation:

The evolution equation for turbulent energy k [22,27]:

(6) $$\frac{\partial \left(\rho k\right)}{\partial t}+\frac{\partial (\rho u_{j}k)}{\partial x_{j}}=\frac{\partial }{\partial x_{j}}\left[\left(\mu +\sigma_{k}\mu_{t}\right)\frac{\partial_{k}}{\partial_{x_{j}}}\right]+P_{k}-\beta^{*}\rho \omega k$$

The evolution equation for energy dispersion ω:

(7) $$\frac{\partial \left(\rho \omega \right)}{\partial t}+\frac{\partial \left(\rho u_{j}\omega \right)}{\partial x_{j}}=\frac{\partial }{\partial x_{j}}\left[\left(\mu +\sigma_{\omega }\mu_{t}\right)\frac{\partial_{\omega }}{\partial_{x_{j}}}\right]+\beta \rho \omega^{2}+2\left(1-F_{1}\right)\sigma_{\omega^{2}}\frac{\partial k}{\partial x_{j}}\frac{\partial \omega }{\partial x_{j}}-\beta^{*}\rho \omega k$$

where

- *k*—kinetic energy of turbulence;
- ω—specific dissipative energy;
- $\mu_{t}$—turbulent viscosity;
- μ—dynamic viscosity;
- $P_{k}$—production of turbulent kinetic energy;
- $\sigma_{k},\sigma_{\omega },\beta ,\beta^{*}$—model constants;
- $F_{1}$—model function.

We should also emphasize the possibility of using another turbulence model, e.g., *k*-ε, but referring to simulation accuracy and computation time, the choice of the proposed turbulence model is justified.

### 2.4. Boundary Conditions

Pressure inlet and pressure outlet were used as inlet conditions. The steam flow through the NP section of the turbine was known to be 80 kg/s, and the outlet pressure value was defined as the pressure prevailing in the condenser.

Outlet pressure was 4.21 kPa; outlet temperature was 29 °C.

In CFD simulations of the last stage of a steam turbine, boundary conditions set as inlet pressure and outlet pressure are often used. These conditions play a key role in mapping the steam flow through the turbine blades and in assessing the energy efficiency of this stage.

Pressure inlet defines the pressure at the steam inlet to the last stage of the turbine. This condition is based on the pressure resulting from the earlier stages of the turbine and is determined by thermodynamic calculations. These calculations take into account the balance of energy flowing through the turbine based on the enthalpy drop across the turbine stages. Thermodynamic analysis uses equations of state, such as the Clausius–Rankine equation [21], to describe the processes in the turbine and the energy balance equation, which determines how much heat is converted into mechanical work. From these calculations, the pressure at the inlet to the last stage of the turbine and other parameters, such as flow velocity, are derived, taking into account mechanical losses and non-reversible heat transformations.

The pressure outlet, on the other hand, defines the pressure and temperature at the outlet of the last stage of the turbine, which are usually known and correspond to the conditions in the condenser. The outlet pressure is usually very low as the steam leaves the turbine and enters the condenser, where it is cooled. Under vacuum conditions, this ci-pressure can be below 0.1 bar, and the outlet temperature is related to the condensation temperature of the steam.

A key aspect in the analysis of steam flow through the last turbine stage is enthalpy drop. This drop refers to the enthalpy difference between the steam state at the inlet and outlet of that stage.

Under ideal isentropic conditions, the enthalpy drop can be calculated as the enthalpy difference without taking mechanical losses into account. However, in an actual steam turbine, the enthalpy drop is less than the theoretical one due to the presence of energy losses such as friction, turbulence, and other non-reversible processes.

The efficiency of the turbine’s final stage is measured as the ratio of the actual enthalpy drop to the isentropic enthalpy drop. Ultimately, enthalpy drop is responsible for the production of useful mechanical work by the turbine, which is crucial for assessing its efficiency and optimizing the operation of the overall system.

Set boundary conditions such as pressure inlet and pressure outlet, combined with enthalpy drop analysis, enable the accurate modeling and simulation of flow processes in the last stage of a steam turbine to optimize its operation and increase energy efficiency.

## 3. Three-Dimensional Scanning

The scanning process took about 2 h, during which time the entire profile of the scapula and its characteristic elements, i.e., the foot, the central stiffening part, and the bandage, were scanned three times.

**Figure 3:**
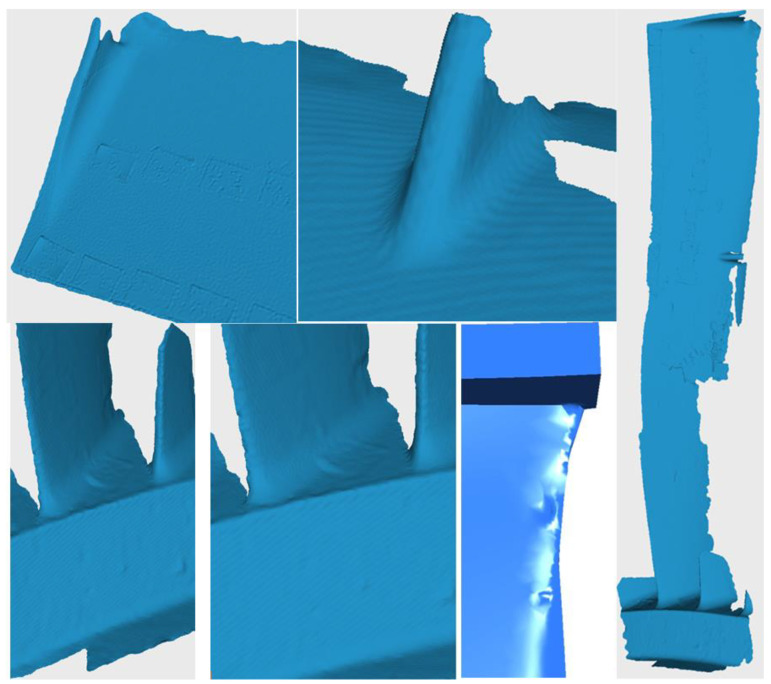
Three-dimensional scans of analyzed blade.

Figure 3 shows the resulting surfaces of the scanned blade.

By taking a large number of scans into account, it was possible to assemble a single geometry, which can be seen in Figure 3.

Although the entire profile was not 100% captured by the 3D scanner, the information on blade length and height and the resulting crosshatching on the profile made it possible to fill in the gaps in the profile.

### 3D Model

This section describes the conversion performed and the addition of planes to the 3D geometry of the blade.

**Figure 4:**
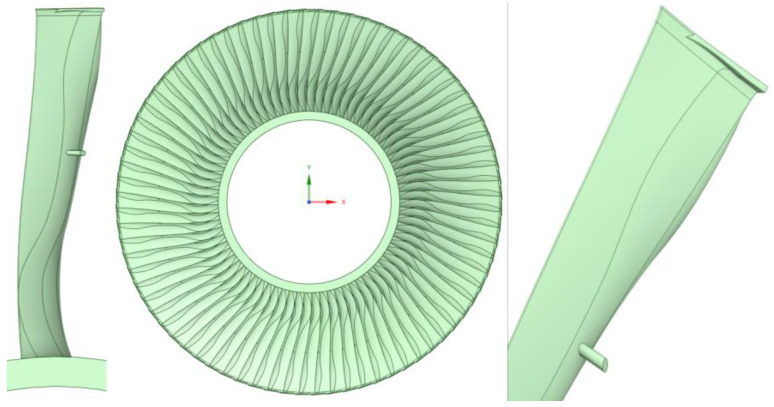
Three-dimensional model of rotor blade and rotor stage.

**Figure 5:**
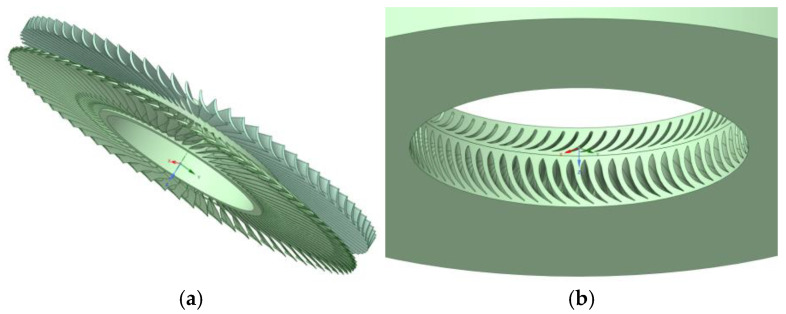
View of the 3D model of the turbine stage. (**a**)—solid domain; (**b**)—fluid domain.

The results of the solid and fluid models are presented in Figure 4 and Figure 5.

First, the scan was imported into a program used to prepare the geometry in Figure 5. The surface was divided into planes, which were then completed on the basis of the information obtained, our knowledge, and the literature to create the 3D geometry of the rotor blade.

The steering vane was made based on photographs taken at the Opole Power Plant site, the literature, and the authors’ knowledge.

The guide vane was selected based on the predicted flows in the turbine stage.

Following the 3D models of the steerable and rotor stage, the fluid domain was spoiled.

The following information was obtained,

- The number of guide vanes—76, number of rotor vanes—76;
- The width and depth of the upper surface of the bead—370 mm × 86 mm;
- The thickness of the banded part of the rotor blade—9 mm;
- The length of the gap between the body and the top of the rotor blade—14.5 mm.

The models do not include the inner body. The fluid model includes the clearance between the body and the top of the rotor blade.

Two geometry models were used to analyze the effect of erosion on flow:

- Actual geometry;
- Geometry smoothed in post-processing.

## 4. Fluid Domain Discretization

**Figure 6:**
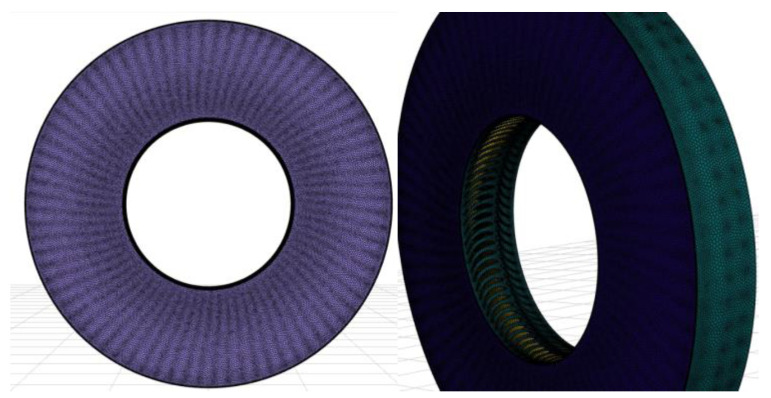
View of the fluid domain from the outlet side of the rotor palisade.

The fluid was discretized using a polyhedral grid. A boundary layer was used. The total number of elements was 12 million, which is shown in Figure 6. Both images in Figure 6 illustrate the polyhedral mesh used in the CFD analysis from different perspectives:

- Left image (outlet view): this shows the mesh from the outlet side, highlighting the mesh structure at the turbine exit, which is crucial for capturing flow separations, pressure losses, and the turbulence caused by erosion.
- Right image (general view): this provides a broader perspective of the fluid domain, showcasing the overall mesh distribution around the turbine blades, and ensuring the accurate resolution of flow features throughout the entire stage.

In summary, both images focus on demonstrating the quality and coverage of the polyhedral mesh, which is essential for accurately simulating the effects of erosion on the turbine’s flow performance.

The fluent’s discretization of the fluid domain involves dividing the computational space into small discrete geometric elements called a mesh. This mesh forms the basis for solving the equations governing fluid flow in the domain using numerical methods such as the Finite Volume Method (FVM) [24,28]. Each grid element corresponds to a small part of the domain, and the flow equations are solved for each element, allowing a distribution of values, such as velocity, pressure, temperature, etc., to be obtained.

The inner body was not included in the 3D modeling and simulations because the focus of this study was on the outer surface geometry and the impact of erosion on the flow dynamics of the rotor blade. The rationale for excluding the inner body is that the primary fluid interactions and erosion effects occur on the exposed surfaces of the blade, particularly the leading and trailing edges, where steam flow causes the most significant changes in performance.

To solve the fluid problem, it was decided to choose a polyhedral mesh with a boundary layer.

Polyhedral meshes are considered to be the most favorable for fluid solutions due to their ability to combine the advantages of both hexahedral and tetrahedral meshes while minimizing their disadvantages. Due to the higher number of faces per element, polyhedral meshes can better represent gradients of variables in the flow, which translates into higher computational accuracy. In addition, the lower number of elements compared to tetrahedral meshes means that calculations can be faster and less resource-intensive while maintaining high-quality results. Better solution convergence and a more uniform distribution of elements make polyhedral meshes often the preferred choice in advanced fluid flow simulations.

## 5. CFD Results

**Figure 7:**
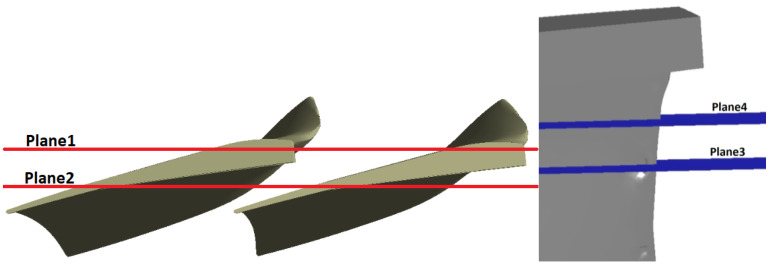
View of Plane 1–Plane 4.

The results are presented in four planes (Figure 7) labeled as follows:

- Plane 1—cross-section through the most eroded edge;
- Plane 2—cross-section in the middle of the flow channel;
- Plane 3—cross-section on the radius 2.12 m (with the most eroded surface);
- Plane 4—cross-section on the radius 2.14 m.

**Figure 8:**
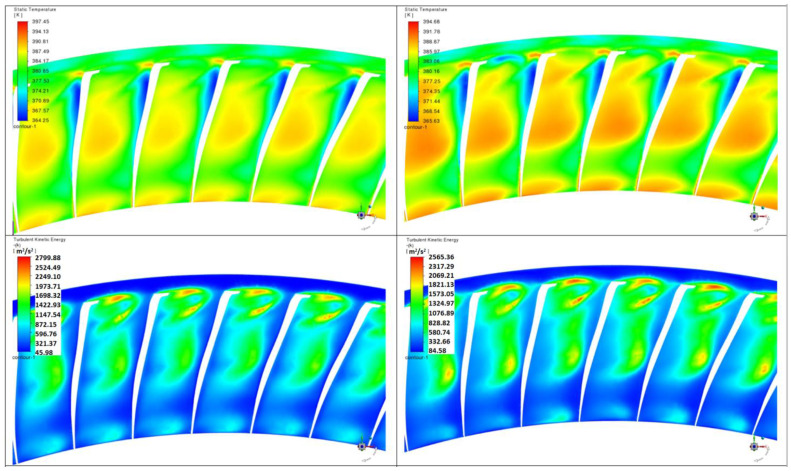
Flow at the leading edge.

**Figure 9:**
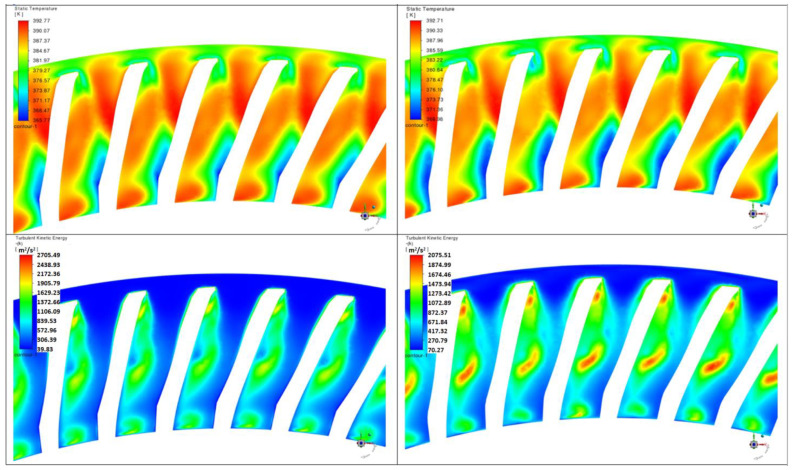
Flow in the middle of the channel.

**Figure 10:**
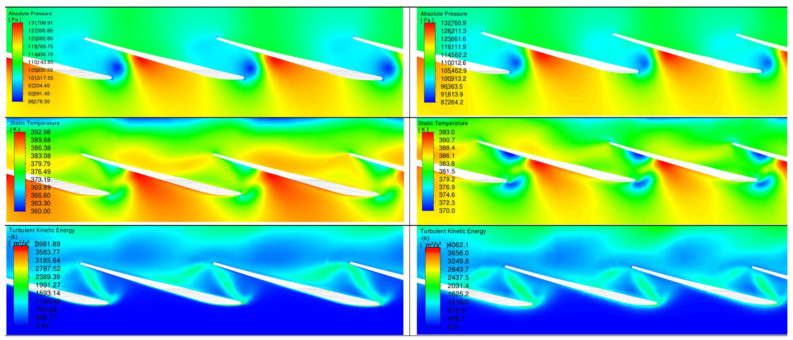
Comparison of flow through a palisade at a radius of 2.12 m before and after erosion.

**Figure 11:**
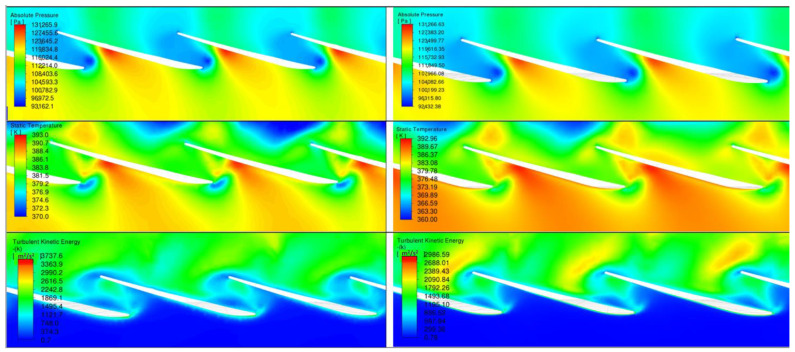
Comparison of the flow through the rotor palisade at a radius of 2.14 m before and after erosion.

Plane 1 and Plane 2 show the temperature and turbulent kinetic energy fields. Plane 3 and Plane 4 show the pressure, temperature, and turbulent kinetic energy fields. In each figure from Figure 8, Figure 9, Figure 10 and Figure 11, on the left-hand side, the results refer to non-zeroed blades and, on the right-hand, side to zeroed blades.

### Non-Eroded Blades

Figure 8 shows that erosion alters the angle of attack and creates larger areas of flow separation in close proximity to the leading edge. The eroded model shows more pronounced turbulence and turbulent flow in areas where there was more orderly laminar flow before erosion.

In the eroded pile in Figure 9, changes in the flow pattern are evident, leading to higher pressure losses and more chaotic flow in the center of the channel. This phenomenon is a direct result of the uneven blade surface and blade damage.

Figure 10 shows the flow for a palisade at a radius of 2.12 m. Erosion, in this case, affects the flow disturbance. The eroded model shows greater flow discontinuities, which can be caused by both blade surface changes and pressure differentials. Larger areas of inflow separation are created on the eroded blades, which increases aerodynamic drag.

Figure 11 shows a clear change in the flow pattern due to erosion. In the eroded model, the reduced smoothness of the flow is observed as a result of the altered blade geometry. There are areas of increased turbulence that suggest flow disturbance associated with the irregularities caused by erosion.

Table 2, Table 3 and Table 4 show the maximum values of the flow parameters at each plane from Plane 1 to Plane 4.

## 6. Discussion

Based on the presented results of the changes in flow parameters and forces in relation to erosion, the following conclusions can be drawn:

For non-eroded blades, the moment of forces was 82,230.8 Nm, while for eroded blades, it dropped to 81,071.0 Nm. This represents a decrease in torque of approximately 0.02%. Erosion reduces the torque, which affects the efficiency of the turbine.

In the case of force, for the non-eroded blades, the value was 79,995.1 N, while for the eroded blades, the value dropped to 79,695.2 N. The decrease in force was relatively small (about 0.038%), which may indicate a less significant effect of erosion on force compared to torque.

An analysis of the changes in flow parameters shows the effect of erosion on three key parameters: temperature, velocity, and turbulence (turbulent kinetic energy).

Temperature: In planes 1 and 2 for eroded blades, the temperature increases compared to non-eroded blades. This occurred on Plane 1 from 397.45 K to 405.41 K and on Plane 2 from 392.77 K to 406.66 K. The increase in temperature may be due to increased internal friction and poorer heat transfer, which compromises efficiency.

Flow velocity: Flow velocity decreased on Plane 1 from 405.41 m/s to 394.68 m/s and on Plane 2 from 406.66 m/s to 392.71 m/s. The decrease in inflow velocity could be related to flow disturbance caused by erosion, which reduces the efficiency of the steam flow.

Turbulent kinetic energy: Erosion clearly reduces the turbulent kinetic energy, which was observed in both Plane 1 (decrease from 2799.88 m²/s² to 2565.36 m²/s²) and Plane 2 (decrease from 2701.94 m²/s² to 2075.51 m²/s²). Lower values of turbulent energy suggest that the flow becomes more orderly; however, this may imply poorer mixing of the vapor molecules, which affects heat transfer efficiency.

Temperature: On Planes 3 and 4, we also observed an increase in temperature for the scoured blades. On Plane 3, there was an increase from 131,708.91 K to 132,760.9 K, and on Plane 4 from 131,266.63 K to 131,265.9 K. The increase in temperature may suggest increased heat loss, which reduces the efficiency of the overall process.

Flow velocity: The velocity on Planes 3 and 4 changed slightly, on Plane 3 from 392.98 m/s to 393 m/s and on Plane 4, from 392.96 m/s to 393 m/s. The effect of erosion on velocity in these planes appears to be negligible, which may be due to the lower impact of erosion on these flow zones.

Turbulent kinetic energy: Turbulent kinetic energy values in Plane 3 increased from 3981.89 m^2^/s^2^ to 4062.1 m^2^/s^2^, indicating a more chaotic flow, while in Plane 4, they decreased from 2986.59 m^2^/s^2^ to 3737.6 m^2^/s^2^. The increased turbulent energy in Plane 3 may imply greater losses due to particle mixing and turbulence, while in Plane 4, the decrease in turbulent energy may suggest a more orderly flow.

## 7. Conclusions

This study analyzed the impact of erosion on ND-45 steam turbine blades in a 900 MW turbine, using 3D scanning and CFD simulations to assess changes in key flow parameters. The following conclusions can be drawn: 

### 7.1. Erosion Impact on Torque and Force

Erosion led to a minor decrease in torque (from 82,230.8 Nm to 81,071.0 Nm) and force (from 79,995.1 N to 79,695.2 N), reducing turbine efficiency slightly. Although the drop in torque was more noticeable, the overall performance impact remained limited after three years of operation.

### 7.2. Changes in Flow Parameters

Erosion increased temperature and decreased velocity across various planes of the blade, indicating reduced heat transfer efficiency. Temperature increases were observed in Planes 1, 2, 3, and 4, while flow velocity decreased primarily in Planes 1 and 2.

Erosion also reduced turbulent kinetic energy on some planes, causing the reduced mixing of vapor molecules, which further affected heat transfer efficiency. In Planes 3 and 4, turbulence increased on one plane but decreased on the other, suggesting variable impacts on different flow zones.

### 7.3. Material Performance

The ND-45 blade material showed good resistance to erosion after three years of operation. Erosion had not yet reached a critical level that would require immediate intervention. The moderate erosion observed indicates that the material remains reliable for continued operation under typical conditions.

### 7.4. Recommendations for Maintenance

Despite the moderate impact of erosion on turbine performance, regular monitoring and maintenance are essential to prevent more severe degradation over time. The early detection of erosion and timely interventions, such as surface hardening or other protective measures, will help extend the blade’s operational life.

In summary, while erosion has a measurable effect on turbine efficiency, the overall performance after three years of operation is acceptable. Continued maintenance and monitoring will be crucial in managing long-term erosion effects.

## Figures and Tables

**Table 1:** Chemical composition of analyzed steel [18].

Chemical Composition	%
Chromium (Cr)	14–15.5
Nickel (Ni)	3.5–5.5
Copper (Cu)	2.5–4.5
Molybdenum (Mo)	0.5
Carbon (C)	Max 0.07
Manganese (Mn)	Max 1
Silicon (Si)	Max 1
Phosphorus (P)	Max 0.03
Sulfur (S)	Max 0.015

**Table 2:** Variation in torque and steam forces in relation to erosion.

Blades	Moment [Nm]	Force [N]
Non-eroded	82,230.8	79,995.1
Eroded	82,071.0	79,695.2

**Table 3:** Change in flow parameters in relation to erosion for Plane 1 and Plane 2.

Blades	Temperature [K]	Velocity [m/s]	Turbulent Kinetic Energy [m^2^/s^2^]
Non-eroded (plane 1)	397.45	405.41	2799.88
Non-eroded (plane 2)	392.77	406.66	2705.49
Eroded (plane 1)	405.41	394.68	2565.36
Eroded (plane 2)	406.66	392.71	2075.51

**Table 4:** Change in flow parameters in relation to erosion for Plane 3 and Plane 4.

Blades	Temperature [K]	Velocity [m/s]	Turbulent Kinetic Energy [m^2^/s^2^]
Non-eroded (plane 3)	131,708.91	392.98	3981.89
Non-eroded (plane 4)	131,266.63	392.96	2986.59
Eroded (plane 3)	132,760.9	393	4062.1
Eroded (plane 4)	131,265.9	393	3737.6

## Data Availability

Data are contained within the article.

## References

[1] Staniša B., Ivušić V. (1995). Erosion Behaviour and Mechanisms for Steam Turbine Rotor Blades. Wear.

[2] Wang S.S., Liu G.W., Mao J.R., He Q.G., Feng Z.P. (2010). Effects of Coating Thickness, Test Temperature, and Coating Hardness on the Erosion Resistance of Steam Turbine Blades. J. Eng. Gas Turbines Power.

[3] Krzyżanowski J. (1991). Erosion of Steam Turbine Blades.

[4] Wen C., Yang Y., Ding H., Sun C., Yan Y. (2021). Wet Steam Flow and Condensation Loss in Turbine Blade Cascades. Appl. Therm. Eng..

[5] Bai T., Liu J., Zhang W., Zou Z. (2014). Effect of Surface Roughness on the Aerodynamic Performance of Turbine Blade Cascade. Propuls. Power Res..

[6] Jiang C., Li W.L., Wu A., Yu W.Y. (2020). A Novel Centerline Extraction Algorithm for a Laser Stripe Applied for Turbine Blade Inspection. Meas. Sci. Technol..

[7] Montis M., Niehuis R., Fiala A. Effect of Surface Roughness on Loss Behaviour, Aerodynamic Loading and Boundary Layer Development of a Low-Pressure Gas Turbine Airfoil. Proceedings of the ASME Turbo Expo.

[8] Pavan A.H.V., Nandi S., Kumar A., Swamy M. (2024). Effect of Laser Hardening and Post Hardening Shot Peening on Residual Stress Evolution in X5CrNiCuNb16-4 Steel for Steam Turbine Blade Applications. Procedia Struct. Integr..

[9] Bai T., Zou Z.P., Zhang W.H., Zhou K., Liu H.X. (2014). Mechanism of Effect of Leading-Edge Geometry on the Turbine Blade Cascade Loss.

[10] Mousavi Anijdan S.H., Moazami-Goudarzi M., Ghohroudi A.N., Jafarian H.R. (2022). Damage Causes and Failure Analysis of a Steam Turbine Blade Made of Martensitic Stainless Steel after 72,000 h of Working. Eng. Fail. Anal..

[11] Azevedo C.R.F., Sinátora A. (2009). Erosion-Fatigue of Steam Turbine Blades. Eng. Fail. Anal..

[12] He Q., Xue S., He H., Hu F., Gao H.C., Hu W. (2023). Fatigue Fracture Failure Analysis of 12Cr12Mo Steam Turbine Blade. Eng. Fail. Anal..

[13] Masoumi S., Lakzian E., Dong Kim H. (2024). Numerical Study of the Impact of Hot Steam Injection in the Condensation Flow through the Low-Pressure Stage of Steam Turbine. Therm. Sci. Eng. Prog..

[14] Oka Y.I., Okamura K., Yoshida T. (2005). Practical Estimation of Erosion Damage Caused by Solid Particle Impact: Part 1: Effects of Impact Parameters on a Predictive Equation. Wear.

[15] Zhou Q., Li N., Chen X., Yonezu A., Xu T., Hui S., Zhang D. (2008). Water Drop Erosion on Turbine Blades: Numerical Framework and Applications. Mater. Trans..

[16] Shulzhenko M.G., Olkhovskiy A.S., Derkach O.L. (2024). The Influence of Kinematic Excitation on the Vibrational Stress of the Mistuned Bladed Disk of the Last Stage of a High-Power Steam Turbine. Procedia Struct. Integr..

[17] Montis M., Niehuis R., Fiala A. Aerodynamic Measurements on a Low Pressure Turbine Cascade with Different Levels of Distributed Roughness. Proceedings of the ASME Turbo Expo.

[18] X4CrNiCuMo14-5 Steel Composition. http://www.ccsteels.com/Stainless_steel/2528.html.

[19] Revopoint POP2 Manual. https://download.revopoint3d.com/support/download/pop2/pop2-quickstartguide-en-v4.0-2023.4.14.pdf.

[20] Markers. https://cadxpert.pl/innowacyjna-technologia-skanowania-3d-zapomnij-o-markerach/.

[21] Badur J. (2005). Five Lecture of Contemporary Fluid Termomechanics.

[22] Bryk M., Głuch J. (2023). A Concept for Safe and Less Expensive Acceleration of a Marine Steam Turbine Start-Up. J. Mar. Sci. Appl..

[23] Badur J., Bryk M. (2019). Accelerated Start-up of the Steam Turbine by Means of Controlled Cooling Steam Injection. Energy.

[24] Kraszewski B., Bzymek G., Ziółkowski P., Badur J. Extremal Thermal Loading of a Bifurcation Pipe. Proceedings of the AIP Conference.

[25] Ziółkowski P.J., Ochrymiuk T., Eremeyev V.A. (2021). Adaptation of the Arbitrary Lagrange–Euler Approach to Fluid–Solid Interaction on an Example of High Velocity Flow over Thin Platelet. Contin. Mech. Thermodyn..

[26] Froissart M., Ochrymiuk T. (2023). Novel Wet Combustion Chamber Concept CFD Studies with Triple Water Inlet. Energy.

[27] Aliabadi M.A.F., Lakzian E., Jahangiri A., Khazaei I. (2020). Numerical Investigation of Effects Polydispersed Droplets on the Erosion Rate and Condensation Loss in the Wet Steam Flow in the Turbine Blade Cascade. Appl. Therm. Eng..

[28] Froissart M., Ochrymiuk T. (2021). Thermal-Fluid–Solid Coupling—Parametrical Numerical Analysis of Hot Turbine Nozzle Guide Vane. Materials.

